# An investigation into the feasibility and efficacy of stereotactic radiosurgery for 1–3 cm single brain lesions on the ring‐mounted Halcyon LINAC

**DOI:** 10.1002/acm2.70105

**Published:** 2025-04-09

**Authors:** Kate Hazelwood, Shane McCarthy, Josh Misa, David Castelvetere, William St. Clair, Damodar Pokhrel

**Affiliations:** ^1^ Medical Physics Graduate Program Department of Radiation Medicine University of Kentucky Lexington Kentucky USA; ^2^ Rhodes College Physics, Biochemistry and Molecular Biology Memphis Tennessee USA

**Keywords:** co/non‐planar geometry, Halcyon LINAC, HyperArc, small brain lesion, SRS, VMAT

## Abstract

**Purpose:**

An evaluation of the accuracy, safety, and efficiency of the Halcyon ring delivery system (RDS) for stereotactic radiosurgery (SRS) treatment to relatively small (1–3 cm) brain lesions.

**Methods:**

After completing the extensive in‐house quality assurance checks including Winston–Lutz test and independent dose verification via MD Anderson IROC SRS head phantom irradiation on Halcyon, fifteen brain SRS patients previously treated with a single dose of 20 Gy on TrueBeam (6MV‐FFF) with HyperArc geometry were retrospectively replanned on Halcyon (6MV‐FFF). Plan quality metrics including conformity index (CI), gradient index (GI), gradient distance (GD), PTV coverage, gross tumor volume (GTV) dose, heterogeneity index (HI), and doses to organs‐at‐risk (OAR) including normal brain dose were evaluated. Patient‐specific quality assurance (PSQA) and independent dose verification via in‐house Monte Carlo (MC) 2nd checks were performed.

**Results:**

The Halcyon was able to provide highly conformal brain SRS plans. When compared to TrueBeam, CI, planning target volume (PTV) coverage, GTV dose (mean and minimum), HI, and doses to brainstem, optic pathway, and cochlea were statistically insignificant. Statistically significant increases in GI (3.76 vs. 3.25, *p* < 0.001), GD (0.56 cm vs. 0.48 cm, *p* = 0.001), and V_12Gy_ (5.5 cc vs. 4.6 cc, *p* = 0.014), on average using Halcyon versus TrueBeam was found, albeit clinically acceptable values for the majority of brain SRS cases. Halcyon plans provided statistically insignificant maximum dose to most adjacent OARs, though there was a statistically significant decrease in the maximum dose to the spinal cord (0.1 Gy vs. 0.4 Gy, *p* = 0.009). Halcyon beam‐on time increases by a factor of ∼2 (*p* < 0.001). However, the faster patient setup on Halcyon results in a comparable estimated overall treatment time for both platforms. Plan deliverability and accuracy was ensured with PSQA (> 95% pass rate for 2%/2 mm clinical gamma criteria) results and MC 2nd check agreement within ± 5.0%.

**Conclusions:**

Halcyon brain SRS plans provided a similar plan quality compared to HyperArc plans, although it demonstrated an inferior intermediate dose fall off thus slightly higher V_12Gy_. This study suggests that Halcyon provides acceptable treatment for solitary relatively small brain lesions of 1–3 cm in diameter. Treatment of select patients on Halcyon will be started at our clinic and it is recommended that other clinics complete an end‐to‐end test, validate, and implement Halcyon SRS treatments at their practices, especially community cancer centers to provide high‐quality service to an underserved patient cohort.

## 1 INTRODUCTION

Brain metastases are a common secondary diagnosis for many cancers. However, recent technological advancements and innovations which potentially leads to better clinical outcomes of brain metastases treatment.^1^ Stereotactic radiosurgery (SRS) has been clinically used as a treatment of brain metastases; when compared with the traditional treatment, whole brain radiotherapy, SRS demonstrated higher overall survival and better local control rate.^1^ In particular, SRS with a single dose of 20 Gy or higher can provide better local control than lower doses as demonstrated by Rades et al.^2^ For SRS treatments of small brain lesions, neurotoxicity is less of a concern^3^; however, there are many challenges to radiation treatments with small field sizes. A primary challenge is the accuracy of the dose calculations, as there are small field dosimetry errors associated with decreasing field sizes.^4^ In addition, the field size is limited to a 1 cm jaw setting by the output factor table on many LINACs, unless it is individually validated and commissioned to smaller than 1 cm field sizes. This presents a challenge with treating small brain lesions on LINAC‐based delivery systems.

Varian Medical Systems (Palo Alto, CA) recently developed the Halcyon v2.0 LINAC, designed to provide fast, effective, and accurate radiation treatments to an underserved patient cohort. The Halcyon ring delivery system (RDS) has a 6MV‐FFF beam and a maximum output of 800 MU/min.^5^ This dose rate is much lower than that of the TrueBeam LINAC, which has a maximum output of 1400 MU/min for 6MV‐FFF beam. The Halcyon RDS has a lower mean beam energy and nominal maximum dose depth (1.3 MeV, 1.3 cm) than TrueBeam (1.4 MeV, 1.5 cm). The beam properties of Halcyon may be beneficial for brain treatment in comparison to TrueBeam, as there is a sharper dose fall‐off associated with the lower mean energy, potentially reducing radiation‐induced toxicity concerns to adjacent organs‐at‐risk (OAR). Additionally, the novel stacked and staggered multileaf collimators (MLC) configuration associated with the Halcyon RDS leads to a low dose leakage and transmission of 0.45%. Halcyon is equipped with a 15‐s iterative kV cone beam CT (iCBCT) image reconstruction that provides high‐quality images for brain treatment.^6^, ^7^ Moreover, this delivery platform includes a one‐step patient setup procedure. The patient is set up using virtual blue lasers and aligning them to skin marks on the patient. Automatic shifts are applied to send the patient to the planned treatment position, then a rapid iCBCT is performed for patient setup and verification. Afterwards, Halcyon automatically applies the residual isocenter shifts for IGRT verification, leading to increased positional accuracy. Despite being limited by three degrees‐of‐freedom (3DoF) couch correction, as reported by Pokhrel et al.^8^ the Halcyon RDS can produce isocenter accuracy (within ± 0.9 mm, per machine specifications) similar to TrueBeam's 6DoF PerfectPitch couch accuracy. This level of positional accuracy is within tolerance for SRS treatment, as recommended by the AAPM TG‐101 report.^9^

There have been a limited number of studies regarding brain SRS/SRT treatments using the Halcyon RDS.^10^, ^11^, ^12^ For instance, Misa et al.^10^ studied Halcyon RDS for relatively large brain tumor beds SRT and provided a satisfactory treatment to one patient in five fractions. Another report by Knutson et al.^11^ revealed similar plan quality and shortened treatment times associated with Halcyon RDS for brain SRT. Their prescription was 30 Gy in 5 fractions for large tumor sizes of 11.1–55.2 cc. Li et al.^12^ performed a retrospective planning study to investigate the utility of Halcyon for treating multiple small brain lesions with a single isocenter. These studies showed comparable plan quality with Halcyon RDS for brain lesions compared to the stereotactic TrueBeam LINAC. To complement these previous studies and commission our Halcyon LINAC for brain SRS/SRT program, first we have done an extensive in‐house quality assurance (QA) checks including routine EPID‐based Winston‐Lutz QA and completed an independent end‐to‐end QA check of dose verification via IROC MD Anderson's SRS head phantom irradiation (IROC, Houston QA Center, TX); and then we retrospectively benchmarked plan quality, delivery accuracy, efficiency, and safety of treating solitary brain lesions (1–3 cm) using a 20 Gy single dose of SRS treatment on the Halcyon v2.0 in our clinical setting. For Halcyon SRS validation, we followed the AAPM TG‐101 recommendations, HyTEC brain SRS guidelines, and the Alliance 071801 brain trial requirements.^9^, ^13^, ^14^, ^15^

## 2 MATERIALS AND METHODS

### 2.1 EPID‐based Halcyon Winston‐Lutz QA test

An in‐house 3D‐printed Winston‐Lutz (WL) Cube phantom (6 cm × 6 cm × 6 cm) with a metallic BB at the isocenter was aligned with the virtual blue lasers and sent to the treatment position on Halcyon RDS. To treat the BB for WL QA test and find the coincidence of radiation and mechanical isocenters, an Eclipse 20‐field 3D‐conformal Field‐in‐Field (2 cm × 2 cm MLC field size center to BB) plan for different gantry, and collimator positions was created with 6MV‐FFF beam. This Halcyon 20‐field equiangular plan covered the entire gantry rotation angles of 360o. This QA plan was delivered through ARIA as parts of Halcyon daily machine QA procedure. The EPID images were then exported from ARIA offline review and analyzed via an in‐house MATLAB script for isocenter positioning accuracy on Halcyon. The average 3D positioning accuracy at isocenter was found to be 0.55 ± 0.30 mm, suggesting acceptable localization accuracy of Halcyon RDS for brain SRS treatment. In addition to the daily machine performance check (MPC), as part of the independent isocenter verification we used this 3D‐printed WL Cube phantom for complementing Halcyon's isocenter positioning accuracy.

### 2.2 Halcyon credentialing for brain SRS

The independent end‐to‐end test, dose verification, and credentialing of the Halcyon brain SRS treatment plan was performed via the IROC MD Anderson's SRS head phantom that containing a 1.9 cm diameter spherical radiosurgical target and dosimetry systems (two thermoluminescent dosimeters, TLD capsules for point dose verification and two orthogonal GAFChromic films for dose profiling) inserted in the anthropomorphic head SRS phantom. Using the departmental SRS guidelines and the instructions provided from the IROC QA center, this head phantom was CT imaged using our brain SRS protocol, planned, and irradiated with an SRS prescription dose of 25 Gy in one fraction to the target (1.9 cm) for credentialing the current ongoing Alliance brain A071801 SRS/SRT trial.^14^ The Halcyon SRS plan contains four full VMAT arcs with 6MV‐FFF beam equipped with stacked and staggered MLC with an effective MLC width of 5 mm at the isocenter. An advanced Acuros‐based dose engine (version 16.1) with a high‐resolution dose calculation grid size (CGS) of 1.25 mm was used in the Varian Eclipse treatment planning system (TPS). Two TLD capsules provided point dose information near the center of the irradiated target. Two orthogonal sheets of GAFChromic film provided dose profiles and an evaluation of the delivered dose distribution through the target, independently. The credentialing results of this end‐to‐end test incorporating dosimetry inserts in the tumor satisfied both the TLD capsules reading and film dosimetric requirements established by the Houston QA center for the brain SRS/SRT treatment on Halcyon LINAC. In this independent dose verification test, the average TLD and film measurement results were within ± 1.0% and 98% gamma index over all three dose planes, respectively. The phantom irradiation results met the MD Anderson's credentialing requirement criteria established by IROC Houston QA Center.

### 2.3 Patient cohort, CT simulation and contouring

After obtaining approval from our institutional review board, 15 previously treated HyperArc brain SRS patients were identified and anonymized. For the original HyperArc brain SRS planning, these patients were immobilized using the Q‐Fix mask, SRS headrest, and mouth bite on the Encompass support device (QFix, Avondale, PA). These patients were set up in the headfirst supine position with arms on both sides of the hand grip array of the Encompass device. High‐resolution 3D axial CT images were acquired via SOMATOM go. Open Pro CT scanner (Siemens Healthineers, Malvern, PA) with a 512 × 512 pixel image size and 1.0 mm slice thickness. This planning CT scan included central and lateral markers on the Encompass device and extended inferiorly to the patient's shoulders to avoid the collision issue for highly non‐coplanar HyperArc geometry. The gross tumor volume (GTV) and OAR delineation previously obtained double contrast‐enhanced high‐resolution MPRAGE MRI scan that was co‐registered to the planning CT in the Varian Eclipse treatment planning system (TPS, version 16.1). The target volumes were delineated by experienced radiation oncologists, defining the GTV by the visible tumor mass in MRI. Per departmental brain SRS protocol, the planning target volume (PTV) was created using a uniform 2 mm expansion margin around the GTV. The relevant OARs delineated for optimization and dose reporting included: the optic pathway, brainstem, spinal cord, cochleae, skin, and brain minus PTV. Tumor locations and characteristics are shown in Table 1. The relatively smaller tumor sizes (1–3 cm) and variety of tumor locations were included to further analyze the future potential of SRS treatments on Halcyon. These patients had varying intracranial tumors ranging from 0.85 to 10.94 cc volume, corresponding to 1.20–2.80 cm in diameter.

**TABLE 1:** Patient characteristics for relatively small brain lesions SRS benchmarking study on Halcyon LINAC.

Patient no.	GTV (cc)	PTV (cc)	PTV diameter (cm)	Tumor location
1	0.25	0.88	1.2	Rt Temporal
2	0.85	2.66	1.7	Lt Occipital
3	5.81	10.63	2.7	Lt Parietal
4	0.34	1.25	1.3	Lt Parietal
5	0.68	1.33	1.4	Rt Parietal
6	2.01	3.44	1.9	Lt Cerebellum
7	0.56	1.87	1.5	Mid Cerebellum
8	1.69	3.77	1.9	Rt Cerebellum
9	0.32	1.05	1.3	Rt Occipital
10	2.55	4.88	2.1	Lt Temporal
11	5.51	9.74	2.6	Rt Parietal
12	4.53	10.94	2.8	Rt Occipital
13	0.34	1.47	1.4	Rt Parietal
14	0.55	1.70	1.5	Lt Frontal
15	0.21	0.85	1.2	Rt Frontal
Mean ± SD (range)	1.70 ± 2.00 (0.21–5.81)	3.80 ± 3.60 (0.85–10.94)	1.80 ± 0.60 (1.20–2.80)	

*Note*: Prescription was 20 Gy in 1 fraction. These patients were treated with HyperArc SRS on TrueBeam. Relatively small tumor sizes of 1–3 cm (in diameter) at different tumor locations in the brain parenchyma.

Abbreviations: GTV, gross tumor volume; PTV, planning target volume.

### 2.4 HyperArc SRS planning and treatment delivery

The clinical HyperArc SRS plans for the smaller intracranial lesions were generated by using the fully automated non‐coplanar HyperArc module on the TrueBeam LINAC with a millennium 120 MLC in Varian's Eclipse TPS (version 16.1). These patients were prescribed a single dose of 20 Gy with the D95% of the PTV receiving 20 Gy or higher per SRS protocol recommendations.^9^, ^13^, ^14^, ^15^ All HyperArc SRS plans had the isocenter automatically chosen and placed within the center of the PTV. After SRS planning, the Virtual Dry Run (in HyperArc Module) was completed to reduce the risk of patient collision with the fully automated delivery system for gantry, couch, and collimator rotations on the TrueBeam LINAC. All HyperArc plans utilized four non‐coplanar arcs of the HyperArc module. To minimize the MLC leakage dose and improve target conformity, the HyperArc module utilized the optimized collimator angles based on the tumor size, shape, and location. The 6MV‐FFF beam (maximum dose rate, 1400 MU/min), SRS normal tissue objective, and entrance beam organ blocking option for both eyes was used. The clinical HyperArc brain SRS plans were calculated using AcurosXB with a fine 1.25 mm dose CGS and tissue heterogeneity corrections.^16^, ^17^ Dose to medium reporting mode was used. As per the brain SRS protocol, target coverage, conformity index, heterogeneity index, gradient index, and maximum dose to OAR constraints including the normal brain dose were reported for all patients. As described before, the brain SRS patients were immobilized via the Encompass support system with a mouthpiece bite. To account for full scatter simulation, the body contour was expanded to include beam attenuation through the Encompass support system.

Before delivering the HyperArc SRS treatment, all physics‐related quality assurance checks including WL, machine QA, and patient‐specific QA tests were completed per departmental protocol following the recommendations laid out by SRS guidelines and AAPM TG‐101 protocol.^9^, ^13^, ^14^, ^15^ On the treatment day, patient setup was performed using our in‐house SRS/IGRT protocol by co‐registering the pretreatment CBCT images with the planning CT scan at the treatment console. Image registration was performed automatically based on bony anatomy within a region of interest, followed by manual refining for more accurate soft tissues matching for optic pathway and brainstem; performed by the treating physician and verified by a physicist. On TrueBeam HyperArc, the brain SRS patient was repositioned via 6DoF PerfectPitch couch corrections before the treatment delivery. The 6DoF couch shifts were within the tolerances of our departmental SRS protocol requirements for all patients. All patients tolerated treatment well.

### 2.5 Halcyon SRS replanning

All HyperArc brain SRS plans were retrospectively replanned using the Varian Eclipse TPS (version 16.1) by an experienced SRS physicist and delivered on the Halcyon LINAC for dosimetric and treatment delivery validation and testing for quality assurance (QA) check. The Halcyon SRS plans with 6 MV‐FFF beam (maximum dose rate, 800 MU/min) utilized four VMAT arcs, mimicking the TrueBeam's arc length and collimator settings but using Halcyon's coplanar geometry. For a more accurate simulation, the Halcyon couch model was inserted. For the Halcyon plans, the MLCs were fitted to the PTV structure and had the initial 3D dose distribution calculated via dynamic conformal arcs (DCA). That dose distribution was used as the starting position for the VMAT optimization. Moreover, Halcyon SRS plans used an MLC aperture shape controller option set to high priority. This novel feature that uses DCA calculated dose before VMAT optimization is available for Halcyon RDS with Eclipse v16.1 or higher versions only. This allows for minimizing the MLC control points with very small openings. For identical prescription and target coverage as clinical HyperArc plans, the different optimization cost functions were used for coplanar Halcyon SRS plans. However, Halcyon plans utilized the same AcurosXB dose calculation algorithm as the clinical HyperArc plans, as well as a fine resolution dose CGS of 1.25 mm with tissue heterogeneity corrections as mentioned above. The average treatment planning time for the Halcyon SRS plans was about 2 h.

### 2.6 Planning goals and plan evaluation metrices

The clinical HyperArc and the Halcyon SRS plans were compared via our departmental brain SRS protocol's criteria for PTV coverage, target conformity index (CI), target heterogeneity index (HI), gradient index (GI), and gradient distance (GD). Defined in Table 2, shown below, is our departmental brain SRS protocol used in evaluating the plan quality for benchmarking the Halcyon SRS delivery platform.

**TABLE 2:** Plan quality evaluation metrics for target coverage, conformity, and intermediate dose fall off for brain SRS treatment applied in our institution.

Parameter	Definition or volume	Clinical goal [acceptable variation]
PTV(D_95%_)	Prescription dose received by 95% of PTV	≥ 20 Gy
PTV(D_98%_)	Prescription dose received by 98% of PTV	—
Min dose to GTV	Minimum dose received by GTV	≥ 20 Gy
Mean dose to GTV	Mean dose received by GTV	—
CI	V100 ÷ PTV	1.0–1.2 [1.0–1.5]
HI	Maximum dose ÷ 20 Gy	1.1–1.4
GI	V_50%_ ÷ V_100%_	< 3.0 [< 4.0]
GD (cm)	Average distance between V_100%_ and V_50%_	< 0.8 cm [< 1.2 cm]

*Note*: This data was summarized from Alliance brain SRS/SRT trial, HyTEC brain SRS papers, AAPM TG‐101 protocol recommendations, and our routine clinical practice.

Abbreviations: CI = conformity index; GI = Gradient index; GD = gradient distance; GTV, gross tumor volume; HI = heterogeneity index; PTV, planning target volume; V_100%_ = volume receiving 100% prescription dose; V_50%_ = volume receiving 50% prescription dose.

All plans on Halcyon RDS were evaluated for the volume of the normal brain receiving 8 Gy (V_8Gy_)–14 Gy (V_14Gy_), respectively. The critical OAR dose metrics were evaluated based on their maximum and volumetric doses as per departmental SRS protocols based on the HyTEC papers, QUANTEC paper, Alliance brain SRS/SRT 071801 trial, and AAPM TG‐101 recommendations.^9^, ^13^, ^14^, ^15^ The dose to OAR and protocol requirements and toxicity concerns are shown in Table 3.

**TABLE 3:** Plan quality evaluation metrics for dose to OAR and the corresponding dose tolerances for single‐fraction brain SRS treatments.

Critical organs	OAR parameter	Clinical goal [acceptable variation]	End point indications [≥ Grade 3 toxicity]
Brainstem	D_max_	< 12.5 Gy [< 15 Gy]	Cranial neuropathy
	D_0.5cc_	< 10 Gy	
Spinal cord	D_max_	< 8 Gy [< 10 Gy]	Myelitis
	D_0.35cc_	< 7 Gy	
Optic pathway	D_max_	< 8 Gy [< 10 Gy]	Neuritis
	D_0.2cc_	< 5 Gy	
Normal brain	V_14Gy_	—	Brain necrosis
	V_12Gy_	< 10 cc [< 15 cc]	
	V_10Gy_	—	
	V_8Gy_	—	
Cochlea	D_max_	< 9 Gy	Hearing loss

*Note*: These values were compiled from Alliance brain SRS/SRT trial, HyTEC brain SRS papers, AAPM TG‐101 recommendations, and from our own daily clinical practice.

Abbreviation: OAR, organs‐at‐risk.

The plan quality metrics for treatment delivery efficiency, total number of monitor units (MU), beam modulation factor (MF = ratio of total number of MU per fraction to the prescription dose in cGy), and beam‐on time (BoT) were recorded. Dosimetric verification of both brain SRS plans was performed on each platform via departmental electronic portal imaging device (EPID)‐based portal dosimetry (PD) patient‐specific QA procedure. The clinical gamma passing criteria for HyperArc and Halcyon VMAT patient‐specific QA were ≥ 95% for 2%/2 mm criteria with a low‐dose threshold set to 10% as similar to the previously reported QA procedures.^18^, ^19^, ^20^ The EPID (aS1200 flat panel detectors, Varian Medical Systems, Palo Alto, CA) device mounted on both TrueBeam and Halcyon LINACs were used. The EPID detector has an active area of 40 cm × 40 cm with a high‐resolution pixel size of 0.34 mm.

Moreover, to verify Halcyon SRS plans independently, the second physics check was performed via an in‐house Monte Carlo (MC) code^21^, ^24^ ^,^ ^2^5; previously implemented in our clinic for routine physics second MU check including small field dosimetry for both Halcyon and TrueBeam HyperArc delivery. This code is based on PENELOPE MC program that utilizes a vendor provided phase space file.^25^ The MLCs were modeled following the schematic drawing provided by the vendor. Briefly, the phase space file contains roughly 2.5 billion particles (photons, electrons, and positrons) per beam configuration. These files were generated by first simulating electron beams in the accelerator using the code package and then transporting through the bending magnet. The electron beam exiting the bending magnet parametrized energy spectrum, spot size, and beam divergence using Gaussian distributions, which were tuned to reference gold beam data.^24^ The detailed description of the clinical implementation and validation of our in‐house MC 2nd check algorithm can be found elsewhere.^24^, ^25^ In our clinical implementation for independent physics second check, to speed up the calculation time, the dose calculation grid size was kept at 2 mm × 2 mm × 1 mm (slice thickness) to achieve a 2% statistical uncertainty. Rather than just evaluating the maximum point dose, in our in‐house MC second check, we have done 3D comparison of the entire distribution and evaluated for the mean planned dose (in Eclipse TPS) for the hottest 1 cc volume around the maximum point dose. Furthermore, overall treatment time was calculated by incorporating the patient setup time, performing pre‐treatment iCBCT imaging, image registration, and applying couch shifts including the dry‐run time (on TrueBeam) on both SRS platforms. The data analysis of dosimetric parameters and plan delivery metrics, including the estimated total treatment times between the two brain SRS platforms, were performed by assessing the normality of each parameter (Shapiro test) followed by an evaluation of skewness and kurtosis.^26^ Comparison of dosimetric parameters and plan delivery metrics was performed using the Wilcoxon rank test (nonparametric) or paired samples *t*‐test (parametric) and a significance level of *p*‐value of < 0.05 via the SPSS 27 data analysis software (IBM, New York, NY).

## 3 RESULTS

### 3.1 Target coverage, conformity, and gradient indices

For both brain SRS plans, plan quality metrics such as tumor dose, PTV coverage, conformity index, gradient distance, and gradient index are shown in Table 4. All plans were in compliance with the recommendations of HyTEC and QUANTEC brain guidelines, alliance brain SRS/SRT 071801 trial, and AAPM TG‐101 recommendations.

**TABLE 4:** Evaluation of plan quality metrics for target coverage, conformity, and intermediate dose fall off for single‐fraction brain SRS on Halcyon LINAC.

Parameter	Clinical HyperArc SRS	Halcyon SRS	*p*‐value
PTV D_98%_ (Gy)	19.92 ± 0.20 (19.52–20.18)	19.92 ± 0.23 (19.59–20.29)	0.649
Min dose to GTV (Gy)	21.46 ± 0.72 (20.02–22.74)	21.34 ± 1.10 (17.82–22.45)	0.625
Mean dose to GTV (Gy)	22.67 ± 0.84 (21.31–23.96)	22.97 ± 0.67 (21.71–24.44)	0.125
CI	1.05 ± 0.06 (0.96–1.16)	1.06 ± 0.05 (0.97–1.16)	0.334
HI	1.18 ± 0.06 (1.08–1.26)	1.18 ± 0.05 (1.10–1.26)	0.549
GI	3.25 ± 0.58 (2.39–4.17)	3.76 ± 0.60 (2.94–4.87)	**<0.001**
GD (cm)	0.48 ± 0.07 (0.37–0.60)	0.56 ± 0.08 (0.47–0.72)	**0.001**

*Note*: Mean ± SD (range) was reported. Statistically significant values are in bold. Prescription dose was 20 Gy in 1 fraction.

Abbreviations: CI, conformity index; GD, gradient distance; GI, gradient index; HI, heterogeneity index; PTV, planning target volume; SD = standard deviation.

**FIGURE 1:**
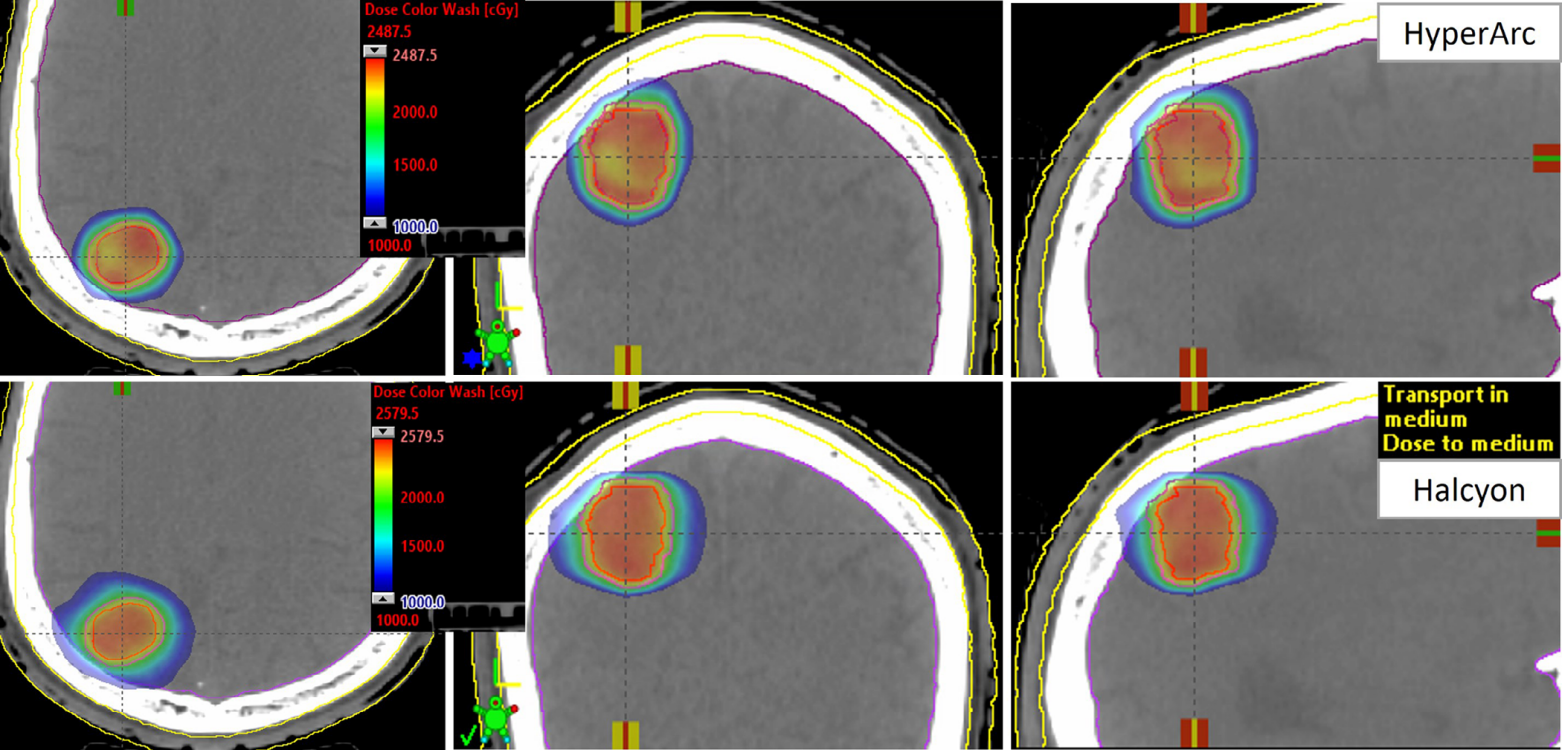
Demonstration of the highly conformal radiosurgical dose distributions for an example patient #11: TrueBeam HyperArc (upper panel) versus Halcyon (lower panel) for right parietal brain lesion. The GTV volume was 5.51 cc. The PTV had a volume of 9.74 cc, equivalent to 2.6 cm diameter tumor size. Isodose color wash with a 50% lower limit is shown for a single dose of 20 Gy treatment plan on both platforms. For similar CIs, due to the Halcyon's coplanar geometry, the GI was higher on Halcyon (3.1) compared to HyperArc (2.4) treatment, but still acceptable per brain SRS protocol requirement.

**FIGURE 2:**
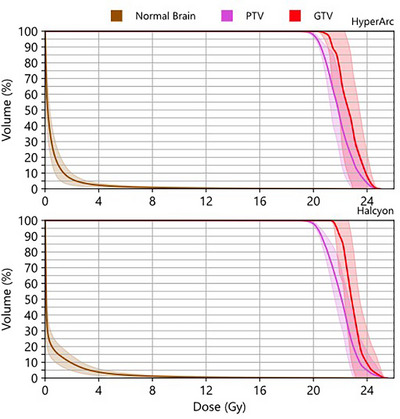
Composite DVHs (GTV, PTV, and normal brain) for the stereotactic single dose on two different treatment delivery platforms: TrueBeam HyperArc (upper panel) versus Halcyon (lower panel). The solid line is the mean value from all 15 brain SRS plans for the corresponding platform, the shadowed region spans ± 1 standard deviation from the mean values. For identical PTV coverage, similar dose to GTVs is seen; however, low dose spread to normal brain was higher on Halcyon.

**FIGURE 3:**
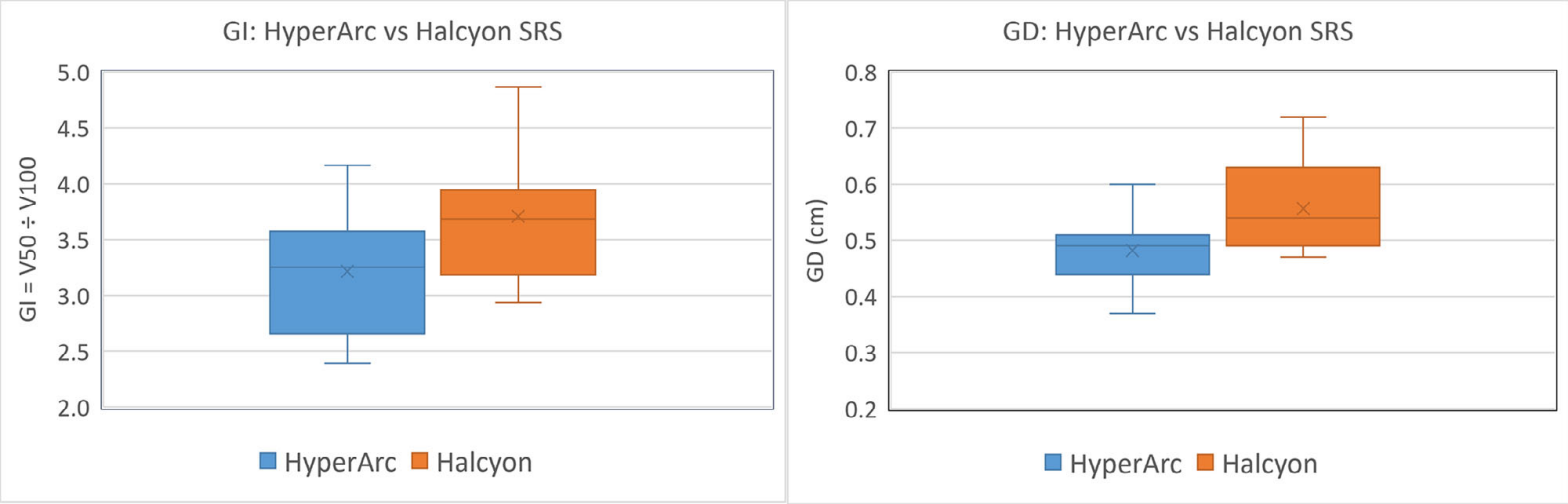
Pairwise demonstration of the mean values and standard deviation of GI and GD for HyperArc versus Halcyon brain SRS plans. Halcyon trailed behind the HyperArc plans for dose gradient parameters, but still able to provide acceptable radiosurgical plans for brain cancer patients.

For the identical PTV D_95%_ coverage, there was no statistically significant difference between the PTV D_98%_. The mean and minimum dose to the GTV between the two SRS plans were similar. In addition to these similarities, the CI and HI had insignificant differences when comparing plans. Both plans demonstrated highly conformal dose distributions, as shown in Figure 1. Though the dose was very conformal, there was a slightly less steep dose gradient in the axial view on Halcyon (see blue isodose color wash). In addition, a higher low dose spread to normal brain tissue can be seen for the Halcyon plans with similar PTV coverage, shown in Figure 2. This indicates an increase in the dose gradient indices (GI and GD) which were found to be statistically significant, as seen in Table 4 and Figure 3. However, they complied with the SRS protocol requirements. The largest significant increase was seen in the GI, which showed a 15.7% increase in the mean value. The statistically significant increase in the gradient indices can likely be attributed to the highly non‐coplanar geometry of the HyperArc compared to the coplanar geometry of the Halcyon. Knowing the value of the gradient distance upfront would allow the planner to make an informed decision on selecting a patient for Halcyon versus HyperArc SRS treatment for achieving the acceptable dose to OAR. Although the mean GI for Halcyon SRS plans was within the clinically acceptable range, there were four plans with a GI above 4.0. Notably, the SRS plans with the highest GI corresponded to the smallest PTVs in the study (< 1.3 cm). This indicates that for very small tumors, if available, the TrueBeam with HyperArc would be a more appropriate treatment modality than the Halcyon due to the higher GI.

### 3.2 Dose to critical organs

Maximum and volumetric doses to the brainstem, spinal cord, optic pathways, and cochlea (maximum) were evaluated. Additionally, the volume of normal brain (brain minus PTV volume) receiving 8 , 10 , 12 , and 14 Gy was evaluated. The results for both plans are presented in Table 5. The Halcyon SRS plans showed statistically insignificant differences in OAR doses to the brainstem, optic pathway, and cochlea. Additionally, there was no statistical difference between the volume of normal brain receiving 10 and 14 Gy, respectively. However, a statistically significant decrease was seen in both the maximum and volumetric dose to the spinal cord with the Halcyon plan, though both plans were within the SRS protocol requirements. As expected, this difference is likely attributed to the coplanar geometry of the Halcyon. In addition, there was a statistical increase in the volume of normal brain receiving 8 and 12 Gy with the Halycon plan, however these differences were small and remained clinically acceptable except for one case (patient #12 with large 10.9 cc PTV size) reported here. This is closely related to the statistically significant increase in the GI, as previously mentioned. Halycon SRS plans were able to effectively spare the OAR with small absolute dose differences.

**TABLE 5:** Evaluation of dose achieved to OAR for corresponding dose tolerances for single‐fraction brain SRS.

Critical organs	OAR parameter	Clinical HyperArc SRS	Halcyon SRS	*p*‐value
Brainstem (Gy)	D_max_	1.7 ± 1.5 (0.40–5.88)	1.7 ± 1.9 (0.04–6.84)	0.695
	D_0.5cc_	1.1 ± 0.8 (0.29–3.38)	1.3 ± 1.2 (0.04–4.29)	0.332
Spinal cord (Gy)	D_max_	0.4 ± 0.4 (0.03–1.68)	0.1 ± 0.1 (0.01–0.19)	**0.009**
	D_0.35cc_	0.3 ± 0.4 (0.03–1.41)	0.1 ± 0.0 (0.02–0.15)	**0.010**
Optic pathway (Gy)	D_max_	0.6 ± 0.5 (0.11–2.24)	0.5 ± 0.8 (0.03–3.1)	0.575
	D_0.2cc_	0.3 ± 0.2 (0.09–0.63)	0.4 ± 0.6 (0.03–2.35)	0.585
Normal brain (cc)	V_14Gy_	3.2 ± 1.5 (1.06–6.10)	3.1 ± 2.2 (0.03–8.10)	0.830
	V_12Gy_	4.6 ± 2.2 (1.56–8.99)	5.5 ± 3.1 (2.18–12.71)	**0.014**
	V_10Gy_	6.6 ± 3.3 (2.25–13.14)	7.6 ± 5.1 (1.01–19.74)	0.259
	V_8Gy_	9.8 ± 5.5 (3.29–19.88)	13.6 ± 8.2 (4.65–31.26)	**0.001**
Cochlea (Gy)	D_max_	0.7 ± 0.6 (0.02–1.75)	0.6 ± 0.8 (0.01–2.33)	0.842

*Note*: See Alliance brain trial, HyTEC brain SRS papers, and AAPM TG‐101 report. Mean ± SD (range) was reported. Statistically significant values are in bold. Prescription was 20 Gy in 1 fraction.

Abbreviation: OAR, organs‐at‐risk.

### 3.3 Treatment deliverability and quality assurance

For both platforms, treatment deliverability was performed for all brain SRS plans by comparing the total MU, beam MF, BoT, and the estimated overall treatment times. Treatment delivery and QA metrics are shown for HyperArc and Halcyon in Table 6.

**TABLE 6:** Comparison of treatment delivery parameters for clinical HyperArc versus Halcyon plans for smaller brain lesion SRS patients.

Treatment and QA Parameters	Clinical HyperArc SRS	Halcyon SRS	*p*‐value
Total monitor units (MU)	5708 ± 1122 (4026–7209)	5609 ± 905 (3686–7163)	0.820
MLC modulation factor (MF)	2.85 ± 0.56 (2.01–3.60)	2.80 ± 0.45 (1.84–3.58)	0.820
Beam‐on time (min)	4.39 ± 0.86 (3.10–5.55)	7.01 ± 1.13 (4.61–8.95)	**<0.001**
Estimated treatment time (min)	11.39 ± 0.86 (10.10–12.55)	12.01 ± 1.13 (9.61–13.95)	0.161
SRS PSQA pass rate [2 mm/2%] (%)	98.6 ± 2.0 (95.3–100.0)	98.9 ± 1.7 (95.5–100.0)	0.483
MC agreement: 2nd physics check (%)	0.6 ± 2.9 (−4.1–4.4)	1.8 ± 1.3 (−0.3–4.2)	0.102

*Note*: Mean ± SD (range) was reported Statistically significant values are in bold.

Abbreviations: MLC, multileaf collimators; PSQA, Patient‐specific quality assurance.

In comparing treatment delivery efficiency, Halcyon and HyperArc plans had similar values for total MU and beam MF. There was a statistically significant increase in beam‐on time for Halcyon. The beam‐on time was 59.7% (2.62 min) higher on average for Halcyon plans due to the lower dose rate (800 MU/min) compared to the 1400 MU/min on TrueBeam, although longer beam‐on time on Halcyon for brain SRS treatment is not desirable. Despite the disparity in beam‐on time, estimated treatment times were similar for Halcyon and HyperArc SRS plans. This is due to the quicker patient set‐up via fully automated “one‐step patient setup” and pre‐treatment verification on the Halcyon, leading to shorter door‐to‐door patient times. Halycon SRS was estimated to take an additional 0.62 min compared to the HyperArc SRS. As mentioned above, treatment delivery accuracy was evaluated by performing the EPID‐based patient‐specific QA and an in‐house MC 2nd check for independent dose verification. There were no statistically significant differences between the PSQA and MC 2nd check, indicating similar treatment delivery accuracy (see Table 6).

## 4 DISCUSSION

In this technical report, we have presented the in‐house WL QA check, independent end‐to‐end test and dose verification via IROC MD Anderson's SRS head phantom irradiation, and dosimetric and treatment delivery study for the feasibility of effective treatment planning and delivery of stereotactic radiosurgery for relatively small brain lesions of 1–3 cm (in diameter) on Halcyon RDS. Our Halcyon has had independent isocenter positional accuracy verification (within machine specifications, < 0.9 mm) through a 3D‐printed in‐house phantom tests; whereas it was comparable to those on TrueBeam (machine specifications, < 0.75 mm) using HyperArc geometry and both plans were compliant with SRS treatment protocol requirements for isocenter positioning accuracy within ± 1 mm. For the target dose and conformity, Halcyon demonstrated slightly inferior gradient indices and V_12Gy_ in comparison to the HyperArc module due to its coplanar geometry. However, these values were still acceptable for SRS treatments; except for one case (patient #12) who had slightly higher V_12Gy_ that may not be acceptable for single treatment for some clinics but can be treated in three treatments, although, for palliative intent that may be acceptable per treating physician's discretion. Moreover, the computation and demonstration of gradient distance on Halcyon for brain SRS could have major implication on patient selection criteria on coplanar Halcyon geometry. For instance, if the brain tumor is 1–3 cm in diameter with nearby critical organs such as the optic pathway (maximum dose < 8 Gy) or brainstem (maximum dose < 12.5 Gy) are at least 1 cm away from the PTV margin, then these patients can be treated with a single dose of SRS on Halcyon RDS. In contrast, a significant decrease in the dose to the spinal cord with the Halcyon system was observed, which was due to not having the vertex non‐coplanar arcs on Halcyon; that may be very useful for retreatment cases. In addition, despite the increase in beam‐on time, there was not a statistically significant increase in the total treatment time, thus completing the SRS treatment on Halcyon in a timely manner.

Recently, there have been a few studies reporting the possibility of creating high‐quality and accurate SRS plans for small fields on Halycon.^10^, ^12^ Similar to our study, Misa et al.^10^ demonstrated the potential utility of Halycon RDS in providing high‐quality SRT treatment for large intracranial tumor beds, like TrueBeam. Their study reported a comparable CI, yet inferior GI for Halcyon SRT plans, similar to this study. However, their study focused on the fractionated treatment of large tumor beds and found a statistically significant difference in HI, which was not observed in our study for a single high dose of radiation to a relatively small solitary brain lesion. Additionally, Li et al.^12^ retrospectively studied small lesion brain SRS plans on TrueBeam and replanned them on Halcyon. They found that the Halcyon design is clinically acceptable for multiple brain lesions with diameter > 1 cm, and that CI was comparable for targets > 1 cm in diameter. Similar to their study, this study found that GI was inferior in Halcyon plans and added a new parameter, GD to further evaluate the SRS plans. However, their study reviewed patients with 6–10 lesions, while this study focused on single lesion. Their multi‐lesion SRS plans would be challenging to execute using a single‐isocenter VMAT SRT plan, as there would be potential spatial uncertainty in patient setup, which could lead to treatment delivery errors on current Halcyon with 3DoF couch. Thus, the first natural step on this new Halcyon LINAC's clinical implementation is to benchmark and validate brain SRS treatment option with single lesion setting (before going to multi‐lesion setting) with isocenter being at the center of the tumor. In addition to daily MPC checks, there is minimal isocenter positioning concern for SRS treatment on Halcyon as we have demonstrated and implemented in our daily WL QA procedure for demonstrating the adequate (within ± 0.9 mm) isocenter positioning accuracy. Additionally, our dosimetric and treatment delivery study using previously delivered HyperArc SRS plans complements the aforementioned reports, as the first step towards for accurately and efficiently treating single and relatively small brain lesions (1–3 cm, in diameter) on Halcyon RDS in the future.

This study demonstrates the potential of treating smaller brain lesions of 1–3 cm on Halcyon with some limitations. First, this study was retrospective in nature, thus it has not been tested on any brain SRS patients on Halcyon yet–this is just a benchmark study of completed end‐to‐end test with dosimetric and treatment delivery tests and validation using actual previously treated HyperArc plans. Future studies will be needed to implement a prospective clinical trial and patient reported follow‐up studies. Another limitation of this study was tumor size. As of now, the field size could not be smaller than 1 cm due to the lack of output factors for less than 1 cm × 1 cm MLC field sizes on our Halcyon. To overcome this situation, ongoing work is being done to collect small field output factors data on Halcyon down to 0.5 cm × 0.5 cm field sizes. Additionally, the current Halcyon RDS has limited couch corrections compared to HyperArc TrueBeam, as rotational adjustments are not currently available on Halcyon. This could potentially lead to patient setup uncertainty, for such high‐precision SRS treatment which have not been dosimetrically quantified, yet. For this preliminary dosimetry study, these Halcyon SRS plans utilized four VMAT arcs, similar to the TrueBeam's arc length and collimator settings but using Halcyon's coplanar geometry. Further optimizing these arcs parameters that could potentially improve the Halcyon SRS plan quality is an interest of further investigation. Moreover, utilizing complex plans with overlapping tumor(s) adjacent to brainstem or Halcyon SRS/SRT planning and validation for more than one brain lesion is warranted.

A few advantages of this brain SRS study on Halcyon are as follows. In the case of longer machine downtime on TrueBeam, a full re‐plan on another machine would be required, resulting in significant treatment course delay. If there is not another stereotactic C‐arm LINAC available in their clinic (like ours), the brain SRS plan can be quickly generated on Halcyon (in about 2 hours) enabling clinic to transfer brain SRS patients between Halcyon and HyperArc, if needed. Halcyon brain SRS program will also be a great option for diverse and big cancer centers with a high SRS/SRT volume, or for patients who require an immediate SRS treatment when other C‐arm LINACs which are not readily available or not beams matched. Furthermore, all Halcyon units around the globe are beam matched thus our brain SRS validation study will be easily reproducible to those clinics with Halcyon, as well as being helpful for underserved community centers with less physics resources to reproduce these key results quickly for SRS program implementation in their clinics.

In this report, we have demonstrated the accuracy, safety, and efficiency of the Halcyon RDS in treating single brain lesions via SRS method. Future studies should provide clinical implementation and report outcomes for select prospective brain SRS patients, as previously mentioned. The field size may also be decreased in the future studies, as the output factor table can be expanded and provide clinically acceptable dose calculation for field sizes down to 0.5 cm × 0.5 cm.^4^ Our ongoing research includes quantifying the dosimetric impacts of rotational setup errors and monitoring intrafraction motion errors to ensure more accurate treatment delivery. Our clinical experience is that metastatic tumor(s) in the brain grow quickly, demanding same day radiosurgery. Similar to our clinical observation, Kutuk et al.^22^ reported changes in tumor diameter, volume, and spatial position in the brain occur as a function of CT simulation to SRS treatment time. In 101 brain SRS patients with a total of 531 brain lesions, they presented ∼10% increase in brain tumor diameter in a week of intra‐scan time interval that was up to 24% for some patients. Thus, the most importantly, our ongoing research includes investigating the potential use of Halcyon RDS for “same day” SRS treatment delivery via RapidPlan model for brain tumors. This would allow for patients to have a treatment planning CT simulation done in the early morning, SRS plan to be generated quickly with the aid of AI‐based auto‐contouring and auto‐planning tools, and then the brain SRS treatment can be delivered soon after following a few QA checks including the EPID‐based PSQA and MC second check, similar to the current Gamma Knife SRS procedure for single and multiple brain lesions.^23^

## 5 CONCLUSION

Our in‐house QA checks, independent end‐to‐end tests, and dosimetric and treatment delivery study demonstrated the high level of positioning accuracy and potential utility of the Halcyon LINAC for SRS delivery of relatively small, 1–3 cm brain lesions, compared to clinically delivered TrueBeam plans with HyperArc. The dosimetric and treatment delivery data collected in this study suggest that Halcyon can deliver safe, effective, and accurate SRS treatments to a small brain lesion. However, as tumor size decreases, GI increases, indicating that very small (< 1.3 cm) tumors may be challenging to treat using Halcyon. Additionally, a patient's door‐to‐door treatment time would remain within the 15 min treatment slot. Treatment of select smaller brain lesions using Halcyon SRS will be starting at our institute. We recommend other Halcyon users to complete their end‐to‐end tests, validate and implement stereotactic treatments on their Halcyon unit, particularly in the community cancer centers and Halcyon‐only clinics. This will expand the outreach of high‐quality stereotactic treatments, including those for patients in underdeveloped countries or remote and underserved patient cohorts.

## AUTHOR CONTRIBUTIONS

Damodar Pokhrel and Kate Hazelwood conceptualized this clinical project. Damodar Pokhrel generated the SRS treatment plans on both platforms. Kate Hazelwood, Damodar Pokhrel, Shane McCarthy, Josh Misa, and David Castelvetere collected and analyzed the data. Damodar Pokhrel and William St. Clair provided radiation oncology clinical expertise and supervision of the paper. Kate Hazelwood and Damodar Pokhrel prepared the preliminary draft of this manuscript. All co‐authors revised, edited, and approved the final manuscript for submission.

## CONFLICT OF INTEREST STATEMENT

The authors declare no conflicts of interest.

## Data Availability

No data available on request due to privacy/ethical restrictions.
